# Time scales in cognitive neuroscience

**DOI:** 10.3389/fphys.2013.00086

**Published:** 2013-04-19

**Authors:** David Papo

**Affiliations:** Center for Biomedical Technology, Universidad Politécnica de MadridMadrid, Spain

**Keywords:** cognitive neuroscience, characteristic time, relaxation time, observation time, non-Gaussianity, scaling, fluctuation-dissipation theorem, non-self-averaging

## Abstract

Cognitive neuroscience boils down to describing the ways in which cognitive function results from brain activity. In turn, brain activity shows complex fluctuations, with structure at many spatio-temporal scales. Exactly how cognitive function inherits the physical dimensions of neural activity, though, is highly non-trivial, and so are generally the corresponding dimensions of cognitive phenomena. As for any physical phenomenon, when studying cognitive function, the first conceptual step should be that of establishing its dimensions. Here, we provide a systematic presentation of the temporal aspects of task-related brain activity, from the smallest scale of the brain imaging technique's *resolution*, to the *observation* time of a given experiment, through the *characteristic time scales* of the process under study. We first review some standard assumptions on the temporal scales of cognitive function. In spite of their general use, these assumptions hold true to a high degree of approximation for many cognitive (viz. fast perceptual) processes, but have their limitations for other ones (e.g., thinking or reasoning). We define in a rigorous way the temporal quantifiers of cognition at all scales, and illustrate how they qualitatively vary as a function of the properties of the cognitive process under study. We propose that each phenomenon should be approached with its own set of theoretical, methodological and analytical tools. In particular, we show that when treating cognitive processes such as thinking or reasoning, complex properties of ongoing brain activity, which can be drastically simplified when considering fast (e.g., perceptual) processes, start playing a major role, and not only characterize the temporal properties of task-related brain activity, but also determine the conditions for proper observation of the phenomena. Finally, some implications on the design of experiments, data analyses, and the choice of recording parameters are discussed.

## Introduction

What's the temporal dimension of cognition? Hardly ever is this question addressed by cognitive neuroscientists, possibly because it appears as either trivial or meaningless.

Cognitive psychologists have long recognized that behavior may often present changes over many time scales (Newell et al., 2001). However, experimental neuroscientists generally make more or less covert assumptions on the dimensions of the phenomena they investigate, some of which become visible when considering the structure of experiments devised to investigate cognitive function. Experiments are typically divided into trials of essentially equal *duration*. On one hand, duration is implicitly equated to the *characteristic length* of cognitive phenomena, with the nested assumptions that such a characteristic length does exist, that it is unique (and therefore stationary modulo learning-related trends), and known *a priori*. On the other hand, duration is equated to the sample mean duration, fluctuations around which are supposed to average out for a sufficiently high number of trials. This set of intertwined covert assumptions also ensures that the phenomenon under investigation is appropriately observed. Furthermore, since each trial is supposed to sample the same aspects of a unique underlying state space, the mere number of trials guarantees that the phenomenon is appropriately observed.

These assumptions hold true to a high degree of approximation for many cognitive (viz. perceptual) processes, but have their limitations for other ones, e.g., thinking or reasoning. For instance, a neuroscientist studying face perception knows the approximate duration of her phenomenon (which indeed has a meaningful *mean duration*) can estimate the number of trials needed to explore the expected cognitive and associated neural range of the phenomenon, and yielding reasonable signal-to-noise ratios. On the contrary, a neuroscientist studying subjects attempting to solve a complicated mathematical problem cannot know what sort of durations (nor, *in general*, what average duration) experiments may yield, what task-related time scales, or quasi-periodicities brain activity may express, and as a result, whether the phenomenon is appropriately observed.

In spite of this unique set of challenging properties, studies of this type of phenomena often apply the same experimental designs, techniques of data analysis, and models of brain activity as those of processes with very different characteristics, e.g., perceptual processes. This, however, supposes some restrictive assumptions, including smoothing response times, to achieve trials of even duration and extract time averages (which are difficult to interpret, given inherent non-stationarities), or using very specific and constrained forms of reasoning (e.g., Goel et al., 1997, 1998; Osherson et al., 1998; Parsons and Osherson, 2001; Bonnefond and Van der Henst, 2009), or limiting the analysis to a very short time window (<1 s), e.g., in the temporal vicinity of an answer to a given problem (e.g., Jung-Beeman et al., 2004; Mai et al., 2004; Lang et al., 2006; Qiu et al., 2008; Pijnacker et al., 2011).

That establishing the temporal scales of cognitive phenomena is both meaningful and fundamental, though, should also become manifest as soon as cognitive function is understood to originate from brain activity, and is quantitatively characterized in terms of the brain properties associated with the execution of given cognitive tasks. Perhaps not so intuitively, in fact, once cognition is measured in units of brain activity, the cognitive processes under investigation are *de facto* treated as physical phenomena. This has two main implications. Firstly, cognition is endowed with the physical dimensions of the associated brain activity. Secondly, the study of cognitive phenomena becomes subject to the same principles and experimental constraints presiding the experimental study of ordinary physical phenomena: dimensions need to be evaluated, through observations of limited time resolution and performed over a finite, often relatively brief, time.

In the following, we systematically review time scale-related aspects of cognition ranging from the *resolution* of the neuroimaging technique, to *observation* time of experiments, and including the characteristic times of the processes under investigation. We discuss some conceptual and experimental implications which should be taken into account when designing experiments in cognitive neuroscience.

## Temporal resolution

Cortical activity helps psychologists refining the space used to describe cognitive function, by adding not only spatial dimensions, but also a much finer graining of the temporal axis, and can therefore help describing cognitive processes even when there is no behaviorally observable event. Thus, it is only logical that experiments should strive to maximize the resolution of the instrument chosen to record brain activity.

Temporal resolution is typically sought for so as to optimize the chances of detecting, and accurately localizing, the onset of events, often lacking a corresponding behaviorally observable one. These landmarks may for instance identify the boundaries between *microstates*, i.e., quasi-stationary segments of duration *l* ≤ 150 ms (Koenig et al., 2002) where the activity field remains stable, punctuated by abrupt changes to new configurations (Fingelkurts and Fingelkurts, 2004; Kaplan et al., 2005). These stable segments were proposed to be “atoms of thought” (Koukou and Lehmann, 1987), supposedly corresponding to different information processing steps.

How well these transitions can be detected does not solely hinge on the instrument used to record brain activity. The instrument's sampling rate sets the lower bound to detectable scales, via the corresponding Nyquist frequency, i.e., half the sampling frequency of a discrete signal processing system. Ultimately, though, the effective temporal resolution of a given experiment is determined by the analyses carried out on the data and, more specifically, by the *size of the smallest temporal fluctuations* that these analyses allow resolving. So, for instance, standard trial-averaging in time-locked evoked potential extraction does not possess millisecond precision, even for a sampling rate of that order, and typically not even that corresponding to frequencies lower than the Nyquist frequency.

The extent to which the boundaries of quasi-stationary segments *per se* can identify a given cognitive process depends on the properties of the phenomenon to be studied.

Event-related perceptual processes are typically modeled as fast phenomena of known characteristic duration *L* ~ 1 s with fluctuations in duration negligible with respect to the mean characteristic duration (δ*L*/〈*L*〉 « 1). For these processes, quasi-stationary segments can be used to partition a given epoch into discrete temporal units, with identifiable temporal landmarks (Kaplan et al., 2005). One can then hope to map the identified landmarks onto identifiable cognitive steps, whose temporal location averaging across repetitions of the same task would then help refining.

A clear partitioning into cognitive steps is more complex for slow processes, such as some forms of conceptual learning, and essentially stationary phenomena, such as memory processes, which often come in episodes with durations various orders of magnitude larger than perceptual processes. A static or comparative statics approach is typically used (e.g., Karni et al., 1995), wherein statical measures of brain activity at different learning steps are compared, ultimately drastically flattening the inherently dynamical aspect of cognition.

Partitioning into cognitive steps processes such as reasoning and thinking is even more difficult, when equipped solely with temporal resolution. Not only do these processes lack a trivial temporal *duration*, but they also come in long episodes (*L* ~ minutes or more), where a large number of cognitive processes interact in a wide range of temporal scales, with unconstrained inner structure and no behavioral correlates most of the time. The associated brain activity is not event-related in the classical sense of the term, as its duration and profile are independent of the physical and statistical characteristics of the stimulus eliciting a given reasoning episode. These processes are also highly non-stationary, rendering the meaning of static descriptions (viz. time averages over long time windows, spanning entire reasoning episodes) problematic.

Thus, for this sort of activity, both the temporal scales of episodes and a partition into cognitive steps become highly non-trivial. The former needs to be evaluated for the underlying physical phenomenon to be properly described, while the meaning of each particular quasi-stationary segment in isolation becomes unclear. As a consequence, extracting meaningful information from a set of different trials of these processes becomes arduous.

In *summary*, we illustrated the concept of temporal resolution, and showed that, rather than by the neuroimaging technique's sampling rate, the effective resolution of a given experiment is ultimately determined by the method used to analyse the data. Temporal resolution is often thought to be necessary and sufficient to identify cognitively relevant quasi-stationary segments of brain activity. This statement holds to a high degree of approximation for fast processes with a typical overall duration, but not for a vast class of cognitive phenomena lacking a typical temporal duration. For this latter class, additional quantifiers of temporal scales need be taken into account.

## Characteristic time(s)

The brain is a non-equilibrium system, exhibiting fluctuations in a wide range of temporal (and spatial) scales, most of which are not observable from behavior alone. Cognition should inherit, though possibly in rather complex ways, a subset at least of the scales of brain activity, which become distinctive properties of cognitive processes.

For short duration perceptual processes, the brain can be modeled as an *excitable medium*, and perception itself is understood as a process whereby the brain relaxes to equilibrium, following exposure to discrete exogenous perturbations or stimuli. For perturbations smaller than a characteristic threshold, a stable stationary solution exists, where the brain is assumed to be quiet on average. Perturbations above the threshold make a dynamical cycle observable, after which the system goes back to its initial resting state, with a characteristic relaxation time of the order of the average duration (τ_relax_ ≈ 〈*L*〉).

On much longer time scales, cognitive processes such as learning may sometimes still be conceptualized as relaxational processes, but the cycle generally takes a non-trivial form. On the other hand, processes such as long-term memory, reasoning and thinking, are respectively stationary and non-stationary non-relaxational processes, and the corresponding neural activity is dominated not by a forcing stimulus, but by endogenous fluctuations (Buice and Cowan, 2009).

These processes have no typical duration *L*, and are generally *temporally extended*, i.e., their duration is much larger than the size of a typical fluctuation (*L* » τ_fluct_). This supposes examining the *time scales* between the temporal resolution and the largest observable one.

A straightforward way to show how the time scales of cognitive tasks arise is to model the associated brain activity as a dissipative dynamic process *X*(*t*), describing the motion of a diffusing macroscopic particle in a complex configuration space. The particle is subject to a viscous friction, changing with a time scale τ_*m*_, and to an additive random force η(*t*) drive having a characteristic time τ_η_ (Hsu and Hsu, 2009). The relationship between τ_*m*_ and τ_η_ determines how microscopic fluctuations renormalize to give rise to observable macroscopic dynamical and statistical properties, and ultimately defines temporal scales of the underlying phenomenon.

Cognitive neuroscience studies typically implicitly adopt a *Markovian approximation*, where the noise has fast vanishing Gaussian δ-correlated fluctuations, and τ_η_ « τ_*m*_. The corresponding process hops without memory from a given configuration to some other, and in the long time limit (*t* » τ_*m*_), the temporal autocorrelation of macroscopic velocity fluctuations *C*_*ii*_(τ) = 〈*v*_*i*_(*x*(τ))*v*_*i*_(*x*(0))〉 (where 〈·〉 designates an average over all times τ) decays as ~ exp(−*t*/τ_*m*_).

τ_*m*_ is unique, and is called *characteristic time*. The Central limit theorem (CLT) ensures that, in the long time limit, the probability density function of the particle's velocity converges to a Gaussian; the mean-square distance (MSD) travelled by the particle increases linearly in time:

(1) $$\langle {\left|x(t)-x(0)\right|}^{2}\rangle \sim t^{2\nu }\text{ with }\nu =\frac{1}{2}$$

a characteristic of normal diffusion.

Note that the relationship between

MSD and autocorrelation function is given by:

(2) $$\begin{array}{l} \langle {\left|x(t)-x(0)\right|}^{2}\rangle =\int_{0}^{t}\int_{0}^{t}\langle \upsilon_{i}(x_{t_{1}})\upsilon_{i}(x_{t_{2}})\rangle dt_{1}dt_{2}\\ \;\approx 2t\int_{0}^{t}C_{ii}(\tau )d\tau \end{array}$$

The characteristic time τ_*m*_, the corresponding correlation time τ_*C*_, i.e., the integral of the autocorrelation function over its support, and the *correlation length*, i.e., the value ξ such that *C*(*t* = ξ) = 0, (with τ_*C*_ = ∫^ξ^_0_*C*(*t*)*dt* ≤ *C*_max_ξ), endow a given process with a temporal scale, independently of its having a characteristic duration, and naturally segment it into separable parts, although in a statistical way.

These scales can be seen as a measure of how fast the system looses memory of its own past. If the system forgets its own past infinitely fast, this time window is infinitely short, and a moment is a point with no dimension. If the system has memory, at each given time, there is an active time window spanning its memory length. This active window can be seen as an estimate of what the system considers as a *moment*.

Furthermore, under the Markovian approximation, spontaneous activity is an unstructured attractor state, and perturbations (e.g., sensory stimuli) produce a temporally local effect, so that what is observed is a *temporally disordered* (*L* » ξ) perfectly elastic fast relaxation process.

In general, the Markovian approximation does not hold when long time scales of brain activity are considered. The friction force becomes retarded or frequency-dependent, and the statistics of the driving noise is neither Gaussian (Freyer et al., 2009) nor δ-correlated. The dynamics incorporates extended memory and temporal non-locality. Ordinary exponential relaxation is replaced by complex, viz. Mittag-Leffler (Bianco et al., 2007a) scaling, with stretched exponential relaxation ~ exp[−(*t*/τ)^α^] at microscopic scales (*t* < τ), and asymptotical convergence to an inverse power law ~ [−(*t*/τ)^α^] for *t* » τ, 0 < α < 1 (Novikov et al., 1997; Linkenkaer-Hansen et al., 2001; Gong et al., 2002; Freeman et al., 2003; Stam and de Bruin, 2004; Buiatti et al., 2007; van de Ville et al., 2010; Freyer et al., 2012). For α ≤ 1, the *correlation time* τ_*C*_ and the corresponding correlation length ξ diverge. Note that correlation time τ_*C*_ and characteristic time τ_*m*_, which coincide for exponential functional forms of the autocorrelation function, do not coincide for power-law ones. The CLT is violated and scale separation is lost, so that microscopic stochasticity becomes detectable at macroscopic scales (Grigolini et al., 1999). Activity undergoes anomalous diffusion with the MSD travelled by the particle no longer a linear function of time:

(3) $$\langle {\left|x(t)-x(0)\right|}^{2}\rangle \sim t^{2\nu }\text{ with }\nu \neq \frac{1}{2}$$

The logarithmic slope of the time-dependent MSD provides an indication of the type of motion: subdiffusion and superdiffusion correspond to $0\leq \nu <\frac{1}{2}$, and $\frac{1}{2}\leq \nu <1$ respectively.

One fundamental implication for the temporal scales of cognition is that activity lacks a characteristic scale, and is described by some relationship defined on a set {τ_*i*_}, rather than by τ_*i*_-s themselves. ℜ can take the form of a scaling exponent ν relating some measure of brain activity to its frequency. Exact self-similarity implies that fluctuations at a given scale are similar to fluctuations at all other scales, so that the probability distribution that the particle has travelled a distance *x* at time *t* is given by:

(4) $$P(x,t)=t^{-\nu }ℱ(x/t^{\nu })$$

where the *scaling exponent* ν is unique for all scales, and ℱ is a *scaling function*. When dealing with real data, self-similarity is not exact but has a statistical sense, and lower and upper cut-offs necessarily appear. Without the scaling range, the scaling properties may be distinctly different from those of Equations 3 and 4 (Latka et al., 2004; Buiatti et al., 2007).

When they exist, the moments of self-similar processes behave as power laws with respect to time (Abry, 2003), and the time dependence of the *q*th moment of the displacement is defined by:

(5) $$\langle {\left|x(t)-x(0)\right|}^{q}\rangle \sim t^{q\nu }\forall q>0$$

Small and large values of *q* sample the central part and the tails of the distribution, corresponding to small and exceptionally large displacements respectively. In real data, the scaling exponent need not be unique for all moments of the distribution, and entire spectrum of scaling exponents may exist. For instance, in the presence of multiplicative interactions and interdependencies among temporal scales (Ihlen and Vereijken, 2010), the moments *q* of the travelled distance may take the form:

(6) $$\langle {\left|x(t)-x(0)\right|}^{q}\rangle \sim t^{q\nu (q)}\nu (q)\neq \text{const}$$

and the underlying diffusion process is weakly self-similar (Ferrari et al., 2001) or strongly anomalous (Castiglione et al., 1999). The motion representing the size of neural events, exhibits steps of all sizes, from local confined motion to extremely long jumps. Furthermore, the scaling need not be of a power-law form (Chainais et al., 2005). The probability density *P*(*x*, *t*) is no longer specified by a unique scaling exponent but by a spectrum of scaling exponents. One way to represent the distribution *P*_λ_(*x*, *t*) at any given scale λ within the scaling range is as the convolution

(7) $$P_{\lambda }(x,t)=G(q,ℜ)⊗P_{\Lambda }(x,t)$$

of the distribution *P*_Λ_(*x*, *t*) at the highest scale and *G*(*q*, ℜ), the probability distribution of the relation across temporal scales (Castaing et al., 1990). More generally, can be seen as a *random process in time scale* τ relating, e.g., the shape of the velocity increment distribution of the dynamic process *X*(*t*) at one time-scale to that at other time-scales (Friedrich et al., 2000; Bacry et al., 2001), or the behavior through scales of the relationship between different models in a renormalization group approach (Longo et al., 2012). For instance, methods have been developed to derive an explicit equation for the changes in the variable *X*(*t*) over a series of nested time-scales of decreasing durations from the data (Friedrich et al., 2011).

The spatially-extended nature of the brain gives rise to additional time scales. Spatial extension introduces not only *dynamical heterogeneity*, i.e., a spatial distribution of time scales, but also time scales emerging from cooperative or competitive phenomena, which typically display regimes and unfold at scales different from those of spatially local components (Bianco et al., 2007b; Allegrini et al., 2011). Space-induced time scales can also stem from temporal non-localities induced by transmission delays, and from transients, whose duration typically scales with the size of the system (Tél and Lai, 2008).

The presence of correlated driving noise and cross-scale relationships produces *temporally ordered* structures (*L* ~ ξ), and dilates a *moment*, from the essentially pointwise extension induced by δ-correlated noise to a temporally *non-local* one, so that in general the meaning of activity at a given time point is not easily divorced from activity occurring within the scaling range.

The size of a moment need not be stationary in time. In fact, the breakdown of exact self-similarity for non-constant values of *q* in Equation 6 implies that there no longer is a unique dilation factor, but a collection of factors with a given distribution. Insofar as temporal scale invariance is a continuous symmetry linking a translation in time to a translation in space, brain activity can be seen as stemming from a system moving at constant velocity, given by the scaling exponent (Sornette, 2004). The break-down of scale invariance (Ciuciu et al., 2011, 2012; Zilber et al., 2012) is tantamount to velocity changes.

One possible way to represent this is to assume that brain dynamics makes steps between given locations of its phase space, in which it dwells for a certain time, and that times between two consecutive steps are characterized by a given *waiting-time distribution* (WTD). The steps mark a sort of internal *operational time*, which can grow sub- or superlinearly with the *physical time*. While in the absence of multiplicative interactions, the function *G* of Equation 7 collapses into a single point and *operational* and *physical* time coincide, multiplicative interactions across scales bias the distribution of waiting times between successive steps. As a result, local probability densities are time-dependent and *intermittent*, with laminar periods interrupted by bursts of large and irregular activity of different sizes (Gong et al., 2007; Freyer et al., 2012).

Intermittency allows reducing brain phenomena evolving in continuous time to event-based point processes and therefore defining the boundaries of individual microstates in a *temporally local* way. Point processes generate events distinct from *background* activity, their value being non-zero only when an “event” occurs, and are thus particularly suitable to describe and model system dynamics characterized by the occurrence of events. The temporal scales of cognitive processes are expressed in terms of the *scaling properties* of the density distribution function *f*(*l*) of microstate durations *l* (Allegrini et al., 2010). *In fine*, if task-related brain activity is considered as a particle evolving in a complex metastable state, the global temporal dimension is of the order of the *Kramers escape time* from or *mean first passage times* in a potential, i.e., the average time elapsed until a stochastic process starting at a given point leaves a prescribed domain for the first time, and typically much longer than the dynamic time scales characterizing states of local stability (Hänggi et al., 1990).

While complex scaling appears as a generic property of spontaneous brain activity, the relevance of the temporal scales and their structure to cognition can be appreciated from two interrelated perspectives.

On the one hand, the stimulus-related dynamic range of a neural network can be related to its spontaneous activity (Shew et al., 2009). This is a consequence of the fluctuation-dissipation theorem (FDT), which establishes a general relationship between the (equilibrium) internal autocorrelation *C*(*t*) of fluctuations of some observable of the system in the absence of the disturbance and the (non-equilibrium) response *R*(*t*) of a system to small external perturbations (Kubo, 1966). Suitably modified versions of the FDT also hold for out-of-equilibrium systems (Cugliandolo et al., 1997; Crisanti and Ritort, 2003; Pottier and Mauger, 2004; Allegrini et al., 2007; Aquino et al., 2007).

On the other hand, the FDT allows conceptualizing cognitive processes as fields acting upon brain activity (Papo, 2013), whose response and associated time scales can be studied using response theory (Kubo, 1966). This conceptualization is particularly intuitive for perceptual stimulus-dependent brain activity. For a generic stimulus-independent process, the effect of cognition can be identified with the noise driving the endogenous dynamics, and asymptotic regimes depend on the ratio between correlation time and intensity of the driving noise, as well as on its statistics (Hänggi and Jung, 1995).

The response to a perturbation depends on the relative time scale of the perturbation and the characteristic times of the system's dynamics. Suppose, for instance, that the brain is a harmonic oscillator of bare frequency ω_0_, and that a given stimulus an applied perturbation asin(ω*t*). Then, additive perturbations would average out over a vanishingly small time interval for ω » ω_0_, would follow the perturbation in a frequency-specific way for ω « ω_0_, and would non-generically show resonance for ω ≈ ω_0_.

However, both ecologic stimuli and brain activity are generally more complex than this simple picture. Accumulating evidence shows that the brain generically responds to changing external fields with a series of avalanches spanning a broad range of scales (Fairhall et al., 2001; Gilboa et al., 2005; Drew and Abbott, 2006; Lundstrom et al., 2008). Fluctuations in ongoing brain activity display *weak ergodicity breaking* (Bianco et al., 2007a; West et al., 2008), a condition wherein the state space is still entirely accessible to the system, but where the *Carlson depth* τ_*Cd*_, i.e., the time that the system requires to visit the whole state space, may be very long, as the system dwells for very long times in some of its accessible microstates.

Cognition may affect cross-scale relations ℜ in a number of different ways: by modulating temporal correlations (Linkenkaer-Hansen et al., 2004; Buice and Cowan, 2009), thereby inducing phase transitions in mean first-passage time regimes (Carretero-Campos et al., 2012); by inducing transitions between non-scaling and scaling regimes or between different asymptotic scaling regimes (Linkenkaer-Hansen et al., 2004; Buiatti et al., 2007; He et al., 2010; Ciuciu et al., 2011, 2012; Zilber et al., 2012), which reflect the cooperative nature of brain activity (Bianco et al., 2007b), and may correspond to dynamical transitions in the system's behavior (Burov and Barkai, 2008); by modulating the degree of non-ergodicity, corresponding to different ways of visiting the state space, a conjecture that has not yet received empirical support. External perturbations may particularly influence the scale at which the WTD is expected to show scaling, while endogenous activity likely affects the scaling properties of the events' waiting time distribution (Aquino et al., 2011). More generally, cognitive function should acquire the temporal scales of the order parameter used to describe it.

All these transitions should ultimately result in operational time and corresponding time scale modulations. For example, plausible testable hypotheses are that cognition may modulate the Markov time τ_*M*_, i.e., the minimum length interval over which the data can be considered as a Markov process, even when the process itself is not Markovian, or the time scale of the transition from microscopic to macroscopic dynamics (Aquino et al., 2007), or the time scales at which fluctuations start converging to a Gaussian distribution (Mantegna and Stanley, 1994; Manshour et al., 2009).

In *summary*, the perceptual neuroscientist deals with an excitable medium, and what is typically studied is a fast linear response to stimulations. It can be treated as an essentially exponential world, with very short memory and a unique characteristic time scale, without losing too much information. The reasoning neuroscientist, on the other hand, faces long time scales of ongoing brain activity, where properties such as non-Gaussianity, non-ergodicity, scale-freeness and long-term memory play a prominent role which cannot be overlooked. A fundamental physical theorem, the FDT, explains why these resting brain properties can be inherited by activity associated with the execution of cognitive task. As a consequence, not only are the time scales of task-related brain activity no longer unique but they also possess complex relationships among them. The time scales and their relationships can be quantified from real data in terms of scaling *exponents* (e.g., of autocorrelation or waiting time distribution functions), scaling *functions* and the typical shape of fluctuations, or even of explicit evolution equations for the position or velocity and for the probability distribution of fluctuations (Fokker–Planck equation). Ways in which time scales of task-independent brain activity can be modulated by task-related brain activity have been suggested.

## Observation time

Cognitive neuroscientists observe phenomena through experiments in which subjects typically carry out a given task a large number of times, which are meant to adequately sample (a meaningful portion of) the phase space of task-related brain activity. Fatigue and other factors limit the typical observation time within a given experimental session to the order of tens of minutes, while experiments (e.g., those assessing sleep-related learning) may consist of more than one session, each of which comprising a given number of trials.

Proper observation of a given process requires that the observation time τ_Obs_ (in this case the entire experiment) be much larger than any scale in the system. Thus, τ_Obs_ should be profoundly different for processes with dissimilar time scales. A measure of observability is given by the *Deborah number D*_*e*_ := τ_rel_/τ_Obs_, i.e., the ratio between the characteristic time of the variable used to describe brain activity, and the length of the time series made available by an experiment (Reiner, 1964). The system is ergodic for small values of *D*_*e*_ but weakly non-ergodic for *D*_*e*_ → ∞ (Rebenshtok and Barkai, 2007).

Various factors at different temporal scales, both within and across trials, may complicate the observability of cognitive phenomena.

The observation time should ideally be much larger than the time needed to visit the phase space of task-related brain activity (τ_Obs_ » τ_*Cd*_). Ensuring sufficient phase space sampling may represent an impervious task when dealing with complex cognitive processes. For instance, if we take the example of a generic open-ended reasoning task, it is difficult to decide whether a reasoning episode, or indeed even an ensemble of episodes, sufficiently sample the repertoire available to a subject. The way the space is sampled by separate trials of an experiment may present some complexity even for fast relaxational processes, where τ_*Cd*_ is approximated by the *Poincaré recurrence time* τ_*P*_. For instance, observed inter-trial fluctuations can be thought of as a sign of the system's exploring its dynamic repertoire (Ghosh et al., 2007; Deco et al., 2011). Contrary to the case of complex forms of reasoning, for which trials can typically be very long, most perceptual tasks are of relative short average duration, and this allows increasing the number of experimental trials.

Another important factor to take into account is *stationarity*, i.e., invariance under time shift of the joint probability distribution, or at least of some moments of the variables quantified in a given experiment.

In behavioral studies (e.g., Ihlen and Vereijken, 2010), single trials are often mapped into a scalar, e.g., a response time, and the process that is considered is given by the sequence of these scalars in the experiment. The underlying cross-scale process is generally considered stationary over the entire experiment encompassing a large number of trials, independently of whether the local probabilities show or not time-dependence.

In cognitive neuroscience, each trial is mapped onto brain activity, and this forces into dealing with within-trial as well as inter-trial (and occasionally inter-session) stationarity. Complex cognitive processes such as reasoning present an inherent dilemma between two opposing needs: to ensure that the tortuous phase space be explored on the one hand, and that the signal be stationary on the other. The former may require extremely long trials. While, in principle, for the inverse power law regime to be observable, trial length needs to be much longer than the time scale of stretched exponential relaxation (Grigolini, 2008), the asymptotic regime may nonetheless be inferred through *finite-size scaling* analysis (Fisher and Barber, 1972). This method takes into account the finite size of experimental data by conveniently rescaling the correlation length ξ by the system's linear size *L* and observing how measured quantities vary for different values of *L*. The values for the critical exponents are then recovered by taking the infinite limit *L* → ∞. On the other hand, in the long time regime, brain activity associated with the execution of complex cognitive tasks is characterized by the presence of long-term memory, weak ergodicity breaking and aging, i.e., temporal correlations and WTDs become dependent on the *observation time* (West et al., 2008). As a result, *D*_*e*_ may diverge and τ_*Cd*_ » 〈*L*〉.

While stationarity may be assured *stricto sensu* only for time scales that are much shorter than the typical duration of, e.g., a reasoning episode, a *quasi-stationary regime* for the susceptibility of the system can be identified over time scales τ_μ_ « τ_QStat_ « τ_rel_ for a given waiting time much larger than the slowest frequency in the data (*t* » ω^−1^) (Crisanti and Ritort, 2003).

A standard assumption in cognitive neuroscience is that separate trials of a given experiment are identically distributed, independent samples, each accounting for the same part of an underlying attractor. The CLT ensures that trial averaged quantities approach a Gaussian distribution. For instance, event-related brain potential studies assume that an observed response *x*_*j*_(*t*) can be decomposed into a stereotyped evoked response *e*_*j*_ occurring with a constant delay after a given stimulus, and additive Gaussian noise ξ_*j*_. Averaging then improves the signal-to-noise ratio by a factor $\sqrt{N}$, where the number of responses *N* typically equals 20–300, and 〈*x*_*j*_(*t*)〉 → *e*_*j*_(*t*) for *N* → ∞. However, under conditions that are explained below, trials may be neither identically distributed, nor independent in practice, so that adding trials may not increase the signal-to-noise ratio as the square root of the number of trials.

For trials to represent a basic periodicity, not only should {τ_*i*_, ℜ} be equal for all trials, in a statistical sense, but it is also necessary that τ_*Cd*_ ≤ 〈*L*〉. If, on the contrary, τ_*Cd*_ > 〈*L*〉, each trial corresponds to different parts of a vast attractor with trial-specific subparts (rather than sampling the same part of the state space). Trial repetition could then improve phase space exploration rather than the signal-to-noise ratio. This may for instance be the case of different reasoning trials of a given experiment, as there is typically more than one way to carry out a given task, and the repertoire of corresponding brain activity may be vast.

Trial independence requires that the inter-trial interval be *ITI* » τ_relax_. For example, in perceptual tasks, ensuring inter-trial independence requires that the brain response to a given stimulus vanishes before the following is presented. This condition may be harder to fulfil than is often assumed in standard event-related potential studies, given that the brain response to stimuli does not vanish exponentially fast, but rather in a history-dependent broad-band fashion (Gilboa et al., 2005; Drew and Abbott, 2006; Lundstrom et al., 2008).

The presence of learning means that the dynamic repertoire of brain activity may change during the course of the experiment. In other words, the phase space landscape itself may evolve, as a consequence of learning. Landscape fluctuations are usually thought to be much slower than those of the system; however, in many contexts, changes may occur on time scales 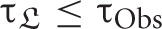 so that the landscape dynamics cannot be neglected. For 
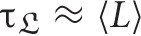
, the statistics of within-trial landscape dynamics can be considered as *quasi-stationary*. When, instead, the landscape shows significant fluctuations at the single trial level 
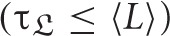
, the phase space itself may not be well-defined.

Within-trial statistics, landscape dynamics, path dependence, and essential transience all affect the way trials of a given experiment can be treated in an aggregate way.

In the presence of scale-free distributions, long-range correlations and inter-trial path-dependence, trials may show weak or no *self-averaging*, i.e., letting the sample become larger does not improve statistics, as dispersion may remain even when the number of trials goes to infinity (Aharony and Harris, 1996). This in turn warns that care should be taken when averaging across trials, and indicates that, at least up to certain scales, the inter-trial temporal scales of a given experiment might better be accounted for in alternative, conceptually different ways, e.g., by characterizing the scaling properties, data collapse and universality of probability distributions (Bramwell et al., 1998; Friedman et al., 2012).

Furthermore, fixed landscape and disorder-averaged properties, respectively corresponding to a distribution of walkers initially concentrated or spread out over the sample (Bouchaud and Georges, 1990) should be treated differently when calculating experiment-wide statistics.

Finally, while relaxation can be slow enough as to *de facto* rule out the existence of an underlying attractor dynamics, different transients can be used to reveal parts of the vector field associated with the cognitive space; estimates of conditional probabilities and of the corresponding stochastic dynamical systems can be derived from multiple, small data sets rather than from of a single long one (van Mourik et al., 2006).

*In summary*, a number of factors may affect observability of cognitive phenomena: sheer complexity of the phase space of task-related brain activity, long-term memory, aging and weak ergodicity breaking of single trial trajectories, path dependence, phase space evolution, and essential transience. These factors influence the way experiments ought to be designed to ensure that the phenomena under study are appropriately observed, the status of single trials of a given experiment, and the corresponding analyses used to aggregate them. Experiments (viz. through their overall length, their design into trials), and analyses (e.g., through thresholds and windowing procedures) both introduce spurious scales of which experimenters should be aware.

## Concluding remarks

A proper characterization of temporal scales provides on the one hand principled guidance for basic experimental steps and, on the other hand, a means to correctly identify the neural correlates of important cognitive phenomena.

Methods of data analysis ranging from the level of inter-trial aggregation procedures down to fundamental single-trial level ones crucially depend on the knowledge of the temporal scales of the phenomenon at hand. Experimental designs should correspondingly hinge on a general understanding of the phenomenon's temporal scales. For example, at the inter-trial level, when studying generic open-ended forms of reasoning, few long trials may ensure better exploration of the state space than multiple time-constrained ones. Short reasoning episodes may have a drastically simpler neural phase space than a single longer one. On the other hand, the longer the considered reasoning episode, the more probable that it accurately visits the complex phase space of task-related brain activity, and the better its time average approximates an ensemble average.

At the single-trial level, measures of brain activity (e.g., local amplitudes, or correlations or synchronization between two recording sites) are often time-averaged within windows in which they are stationary. While the size of time windows is often chosen once and for all for entire data sets, searching for true microstates, which can be thought of as stationary segments, implies a data-driven segmentation (van de Ville et al., 2010). In the presence of long-range temporal correlations, however, defining the boundaries of stationary segments is an inherently arduous task, and even more so when evaluating global microstates of whole-brain activity. On the other hand, ensuring stationarity by considering short time-windows may come at the price of sacrificing temporal scales, as typical procedures *de facto* constitute high-pass filters on the data, and would thus lead to missing key aspects of the dynamics. As a consequence, an appropriate strategy may require working with quantities evaluated over extremely short time-windows (e.g., Bianco et al., 2007a).

A similar conclusion holds for the choice of recording parameters, viz. sampling rates. For instance, surprising though it may read, temporal resolution is in general not sought to increase the ability to understand the complex relationship between processes unfolding at different temporal scales. Indeed, it is fair to say that the very concept of *temporal scale* is essentially alien to standard cognitive neuroscience. A telltale of this is that, curiously, the need for temporal *precision* is often thought to be inversely proportional to the typical temporal duration of the cognitive process to be investigated, rather than to its complexity. In other words, temporal precision is often thought to be needed more when studying short (e.g., perceptual) than long (e.g., in reasoning) processes. The idea is that temporal precision is essentially required to capture fast, fleeting processes, which would otherwise go unnoticed. It is however important to see that, in the case of a complex process such as reasoning, the reconstruction of the tortuous phase space trajectory improves with the amount of available (non-spuriously correlated) time points.

The explicit handling of the temporal dimension of cognition allows framing the neural correlates of given cognitive processes in terms of temporal scales, and their set of relationships. In fact, describing cognitive function may sometimes essentially boil down to specifying {τ_*i*_, ℜ}, which may in turn constitute a necessary condition for the statistical treatment of experimental data. A particularly clear illustration is represented by complex phenomena such as conceptual learning or reasoning and thinking, which, as we have seen, lack both an average temporal duration and inner segmentation. Deriving generic characteristics of such processes in spite of their inherent variability, or evaluating whether a given observation time is sufficient cannot be done without characterizing time scales and describing task-modulated changes in the relationship among processes unfolding at different time scales.

Overall, some general experimental indications for cognitive scientists can be derived. Accounting for the temporal scales of complex cognitive processes may involve changes in the ways experiments are designed, data analysed and brain recording instruments and the relative parameters are chosen. Which of these aspects needs to be changed with respect to standard procedures depends on the type of cognitive process under study. While studying fast perceptual processes may only involve changes in data analysis methods, studying phenomena such as thinking or reasoning may require a more global change of all of these aspects. Indeed, these fundamental changes are necessary to tackle the most challenging aspects of these extremely complex cognitive processes.

Specifically, we highlight four main experimental implications. (1) With phenomena lacking a clear characteristic duration, e.g., open-ended forms of reasoning, it may be interesting to build experiments with few long trials with rich dynamical repertoire. (2) Different trials of a given experiment (be they short-lived perceptual or long duration reasoning tasks) should not automatically be treated as independent samples of the same part of an underlying attractor, and may not self-average as their number is increased. This may bear important consequences both on the type of metric that can be used to quantify brain activity and on the statistical tests that may highlight differences between experimental conditions. (3) At shorter time scales, there is a mutual influence between the time scales of the process and the choice of the window size in which relevant quantities (the choice of which is not the goal of the present article, and depend of the investigator's goals) are calculated. Decisions must then be made as to the size of these windows. (4) While an appropriate temporal sampling rate is necessary to detect abrupt short-lived transitions, irrespective of the considered cognitive phenomenon under study, it is also necessary to reconstruct the underlying dynamics, contrary to the common stance whereby processes such as reasoning or thinking may just need a poor time-resolution recording device.

Finally, at a more conceptual level, the time scales of a given phenomenon and, in particular, the relationship ℜ among them contain information on the mechanisms through which cognitive processes interact to give rise to cognitive performance (Holden et al., 2009). The long time limit loss of time-scale separation provides a possible mechanism through which changes at faster time scales (e.g., fast perceptual and attentional processes) may be embedded into activity (e.g., memory processes) unfolding at times scales several orders of magnitude larger (Fujimoto and Kaneko, 2003). The presence of scaling in a broad range of time scales suggests that observable behavior is but the uppermost level of a scale-invariant phenomenon and may be understood as a macroscopic coarse-grained aspect of ongoing brain activity (Fingelkurts et al., 2010), resulting from the renormalization of its fluctuations, and that cognitive function may not always be decomposable into less complex component processes. Time can also be regarded as a variable revealing the constructive rules underlying the *spatial* structure of task-related brain activity at various scales (Pérez-Mercader, 2004; Itzkovitz et al., 2005; Delvenne et al., 2010).

### Conflict of interest statement

The author declares that the research was conducted in the absence of any commercial or financial relationships that could be construed as a potential conflict of interest.
